# A Nomogram for Predicting Prognosis of Advanced *Schistosomiasis japonica* in Dongzhi County—A Case Study

**DOI:** 10.3390/tropicalmed8010033

**Published:** 2023-01-03

**Authors:** Zhong Hong, Shiqing Zhang, Lu Li, Yinlong Li, Ting Liu, Suying Guo, Xiaojuan Xu, Zhaoming Yang, Haoyi Zhang, Jing Xu

**Affiliations:** 1 National Institute of Parasitic Diseases, Chinese Center for Disease Control and Prevention (Chinese Center for Tropical Diseases Research), NHC Key Laboratory of Parasite and Vector Biology, WHO Collaborating Centre for Tropical Diseases, National Center for International Research on Tropical Diseases, Shanghai 200025, China; 2 Department of Schistosomiasis Control and Prevention, Anhui Institute of Parasitic Diseases, Hefei 230061, China; 3 Department of Clinical Treatment, Dongzhi Schistosomiasis Hospital, Chizhou 247230, China

**Keywords:** advanced schistosomiasis, prognosis, LASSO logistic regression, nomogram

## Abstract

Backgrounds: Advanced schistosomiasis is the late stage of schistosomiasis, seriously jeopardizing the quality of life or lifetime of infected people. This study aimed to develop a nomogram for predicting mortality of patients with advanced schistosomiasis japonica, taking Dongzhi County of China as a case study. Method: Data of patients with advanced schistosomiasis japonica were collected from Dongzhi Schistosomiasis Hospital from January 2019 to July 2022. Data of patients were randomly divided into a training set and validation set with a ratio of 7:3. Candidate variables, including survival outcomes, demographics, clinical features, laboratory examinations, and ultrasound examinations, were analyzed and selected by LASSO logistic regression for the nomogram. The performance of the nomogram was assessed by concordance index (C-index), sensitivity, specificity, positive predictive value (PPV) and negative predictive value (NPV). The calibration of the nomogram was evaluated by the calibration plots, while clinical benefit was evaluated by decision curve and clinical impact curve analysis. Results: A total of 628 patients were included in the final analysis. Atrophy of the right liver, creatinine, ascites level III, N-terminal procollagen III peptide, and high-density lipoprotein were selected as parameters for the nomogram model. The C-index, sensitivity, specificity, PPV, and NPV of the nomogram were 0.97 (95% [CI]: [0.95–0.99]), 0.78 (95% [CI]: [0.64–0.87]), 0.97 (95% [CI]: [0.94–0.98]), 0.78 (95% [CI]: [0.64–0.87]), 0.97 (95% [CI]: [0.94–0.98]) in the training set; and 0.98 (95% [CI]: [0.94–0.99]), 0.86 (95% [CI]: [0.64–0.96]), 0.97 (95% [CI]: [0.93–0.99]), 0.79 (95% [CI]: [0.57–0.92]), 0.98 (95% [CI]: [0.94–0.99]) in the validation set, respectively. The calibration curves showed that the model fitted well between the prediction and actual observation in both the training set and validation set. The decision and the clinical impact curves showed that the nomogram had good clinical use for discriminating patients with high risk of death. Conclusions: A nomogram was developed to predict prognosis of advanced schistosomiasis. It could guide clinical staff or policy makers to formulate intervention strategies or efficiently allocate resources against advanced schistosomiasis.

## 1. Introduction

Human schistosomiasis is a water-borne infectious disease caused by blood flukes of the genus *Schistosoma*. The disease occurs worldwide in 78 countries and regions in Asia, South America, the Middle East, and Africa. Globally, over 780 million people are at risk of infection, and 250 million have been infected with *Schistosoma* spp., of which 90% are concentrated in sub-Saharan Africa [1,2,3]. The estimated global burden of schistosomiasis is 3.31 million disability-adjusted life years (DALYs) [4]. There are three main species of schistosomes infecting human beings: *Schistosoma japonicum*, *Schistosoma mansoni,* and *Schistosoma haematobium* [2]. The former two species cause intestinal schistosomiasis and impair the liver, spleen, and/or intestinal tissues. The symptoms present as nonspecific intermittent abdominal pain, diarrhea, rectal bleeding [5], periportal fibrosis [6], portal hypertension, ascites, and hematemesis [7]. *S. haematobium* causes urogenital schistosomiasis, leading to pathological effects such as hematuria, chronic fibrosis of the urinary tract, hydroureter, hydronephrosis [8], and genital tract damage [9]. Among the three major human schistosomiasis, schistosomiasis japonica often results in more severely pathological lesions than the other two schistosomiasis due to the more significant ova production of adult *S. japonicum* [10].

China used to be an endemic country, carrying the heaviest disease burden of schistosomiasis japonica [11]. To provide guidance for clinical staff to treat patients efficiently, schistosomiasis japonica was categorized as acute, chronic, and advanced schistosomiasis based on history of patients exposed to infested water with cercaria of schistosomes, results of laboratory examination, and clinical symptoms [12,13]. Advanced schistosomiasis japonica is the extreme form of schistosomiasis japonica with high mortality before Praziquantel was available. It is often associated with severe growth retardation, spontaneous bacterial peritonitis, refractory ascites, frequent bleeding of the upper gastrointestinal tract, hepatic failure, etc. [14]. As the morbidity and prevalence of schistosomiasis has decreased significantly after 70 years’ efforts, the Chinese government has paid more attention to advanced schistosomiasis due to the lack of an efficient therapeutic approach and poor prognosis [15,16]. In 2021, there were 29,037 cases of advanced schistosomiasis in China, and 1526 advanced cases died from this disease in that year [17]. Since early intervention is associated with improved prognosis [18], identifying patients at high risk of death will ensure that these patients receive appropriate treatment and long-term follow-up. Therefore, it is essential to develop a clinical tool that can help clinics to identify patients with advanced schistosomiasis of high mortality risk accurately and provide guidance for management decisions to decrease the disease burden. 

Accurate prognostic evaluation is the basis of prevention and treatment, in which clinical prognostic factors must be clearly illustrated [19]. Survival analyses, such as the Cox proportional hazards model (CPH), were used to find the impact factors of clinical prognostic outcomes in clinical research, but it may be too simplistic for some complex clinical events, such as progression to death. However, the development of machine learning has enabled predictive models to be applied further in many medical kinds of research [20,21,22,23]. For example, the nomograms have made prognoses quickly understood and have helped clinical decision making through rapid computation in visualized user interfaces [24]. They have recently been widely applied for predicting prognosis in cancers [25], acute infectious diseases [18,26], and chronic diseases [27,28]. Predictive models in limited studies were constructed by univariate–multivariate analysis based on relatively few variables and small sample size [19,29]. Given increasing values of multiple variables, a more comprehensive and personalized prognostic model is necessary for advanced schistosomiasis. In this study, we conducted a population-based study using clinical data combined with machine learning arithmetic to develop a nomogram for predicting prognosis of patients with advanced schistosomiasis japonica.

## 2. Methods

### 2.1. Data Source and Study Population

Dongzhi County (Supplementary Figure S1), located in the south of Anhui Province of China along the Yangtze River, is an endemic county of schistosomiasis japonica with both ecotypes of hill and marshland settings. More than 800 patients with advanced schistosomiasis japonica are enrolled in the county, and about 35 patients die each year. According to the Diagnostic Criteria for Schistosomiasis (WS261–2006) issued by China’s Ministry of Health, the patients who met the following four criteria were confirmed as having advanced schistosomiasis: (1) they had ever lived in endemic areas of schistosomiasis japonica and had a history of exposure to *S. japonicum*; (2) they presented clinical symptoms such as ascites, splenomegaly, portal hypertension, and gastroesophageal variceal bleeding, or with granulomatous lesion of the colon and rectum or severe growth retardation; (3) they were examined as anti-*S. japonicum* antibody-positive; (4) they were pathologically positive, as tested by stool examination or rectal biopsy. 

Data reflecting demographic, clinical, laboratory, and ultrasound features of advanced patients admitted to Dongzhi Schistosomiasis Hospital were collected from January 2019 to July 2022. Patient identity information is kept strictly confidential. Moreover, the patients were granted the right to waive participation without adversely affecting their rights and benefits.

### 2.2. Inclusion and Exclusion Criteria of Participants

The inclusion criteria of patients were as follows: (1) patients agreed to participate in this study; (2) patients had completed information, including demographic and clinical information; (3) patients were diagnosed correctly; (4) patients met the criteria of China’s treatment and assistance programs on advanced schistosomiasis japonica.

The exclusion criteria of patients were as follows: (1) patients refused to participate in this study; (2) patients had missing information, including a lack of population-based demographic, clinical, laboratory, ultrasonic data, and survival outcome; (3) patients with other diseases whose symptoms were as similar as advanced schistosomiasis, including primary hepatocarcinoma, primary hypersplenism, primary ascites, and primary liver fibrosis; (4) patients had not been included in China’s treatment and assistance programs on advanced schistosomiasis japonica.

### 2.3. Candidate Variables for Prediction

There were 34 variables included: survival outcome (death or not), demographic data (age, gender, occupation), clinical data (splenectomy, cholecystectomy, hypertension, hypoalbuminemia, hypokalemia, gastrointestinal bleeding, coagulopathy, diabetes, hepatic encephalopathy, anemia level, body mass index (BMI)), laboratory data (HBV infection, aspartate aminotransferase/alanine aminotransferase ratio (AST/ALT), albumin (ALB), total protein (TP), albumin/globulin ratio (A/G), creatinine (CREA), high-density lipoprotein (HDL), CA-125 antigen (CA-125), hyaluronate (HA), laminin (LN), N-terminal procollagen III peptide (PIIIPN-P), IV collagen (CIV), total bilirubin (TBIL), direct bilirubin (DBIL)), and ultrasonic data (liver fibrosis level, atrophy of the right liver, gallbladder disease). The patients were followed up to the study’s deadline, or the death occurred. Positive outcomes in this study were death occurring during hospitalization and after discharge.

### 2.4. Establishment of Training Set and Validation Set

The patients were randomly divided into training and validation sets with a ratio of 7:3 to ensure the distribution of outcome events and factors without significant difference between the two datasets. The training set was used to screen the predictors and construct the model. The internal validation set was used to evaluate the model performance.

### 2.5. Model Derivation

We used SPSS version 25.0 (SPSS, Chicago, IL, USA) and R software version 5.0 (https://www.r-project.org, accessed on 2 July 2022) to conduct the statistical analysis.

Descriptive statistics were used to analyze the baseline information in model derivation and internal validation. Differences in categorical variables were assessed using the chi-squared test. All *p* values were two-tailed, and *p* < 0.05 was considered statistically significant. Penalized regression was used to select relevant features regarding the death probability of patients by the “glmnet” package of R. Penalized regression is recommended by the transparent reporting of a multivariable prediction model for individual prognosis or diagnosis (TRIPOD) checklist for developing and validating risk and diagnostic models [30]. Regularization is a technique that adds a penalty to the objective function. This penalty controls the model’s complexity by shrinking the regression coefficients’ values. If the shrinkage is exactly zero, it is then called the L1 norm or L1 penalty [31,32]. The least absolute shrinkage and selection operator (LASSO) uses L1 penalties. The penalty term (λ) is controlled by a regularization parameter (k), which was selected using a cross-validation procedure [33]. In this study, k was chosen using threefold cross-validation [33]. We constructed a logistic regression model based on the candidate predictors screened by the LASSO regression. We selected the final predictors based on the Akaike information criterion using the backward selection approach. Meanwhile, the variance inflation factor (VIF) was assessed among the variables, and VIF > 4.0 was interpreted as indicating multicollinearity. Variables with VIF > 4.0 were excluded from the final model analysis.

### 2.6. Assessment of Model Performance

Performance of the established model was evaluated in the following ways: (1) Sensitivity, specificity, positive predictivity value (PPV), and negative predictivity value (NPV) were calculated to evaluate the performance of the model. (2) Concordance index (C-index), which was equal to the area under the receiver operating characteristic curve (ROC) in binary logistic regression [24], was calculated by bootstrapping (1000 resamples) to evaluate discriminative ability. The C-index varies from 0.5 to 1.0, where 0.5 represents random chance, and 1.0 indicates a perfect fit. Typically, C-index and AUC values larger than 0.7 suggested a reasonable estimation [34]. (3) Calibration plots were used to evaluate calibrating ability. Typically, the calibration curve was close to the ideal curve, suggesting that the model fitted well. (4) Decision curve analysis (DCA) was used to evaluate the nomogram’s clinical net benefits and utility. DCA is a method for evaluating the clinical benefit of alternative models and was applied to nomograms by quantifying net benefits at different threshold probabilities [34]. The curves of the treat-all-patients scheme (representing the highest clinical costs) and the treat-none scheme (representing no clinical benefit) were plotted as two references [35,36].

## 3. Results

### 3.1. General Information of Patients

**Figure 1:**
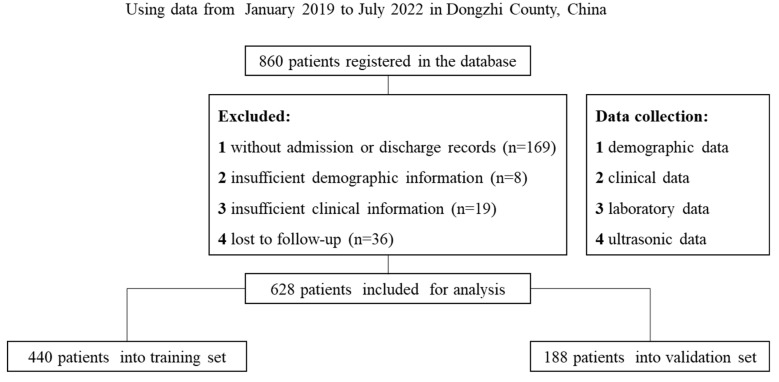
The flowchart illustrates this study’s procedure, including the study population selection, data collection, and the basis of the training and internal validation sets.

Of the 860 patients registered in the database, 762 met the inclusion criteria and were included in the final analysis, with 440 assigned to the training set and 188 to the internal validation set randomly (Figure 1). No significant difference was detected in any variable between the training set and the internal validation set (*p* > 0.05) (Table 1). There were 185 males and 255 females in the training set, with 196 of them ≤65 years old and 244 patients > 65 years old. A total of 94 males and 94 females were divided to the validation set, with 86 patients ≤ 65 years old and 102 > 65 years old.

### 3.2. Risk Factors Affecting Outcomes

**Figure 2:**
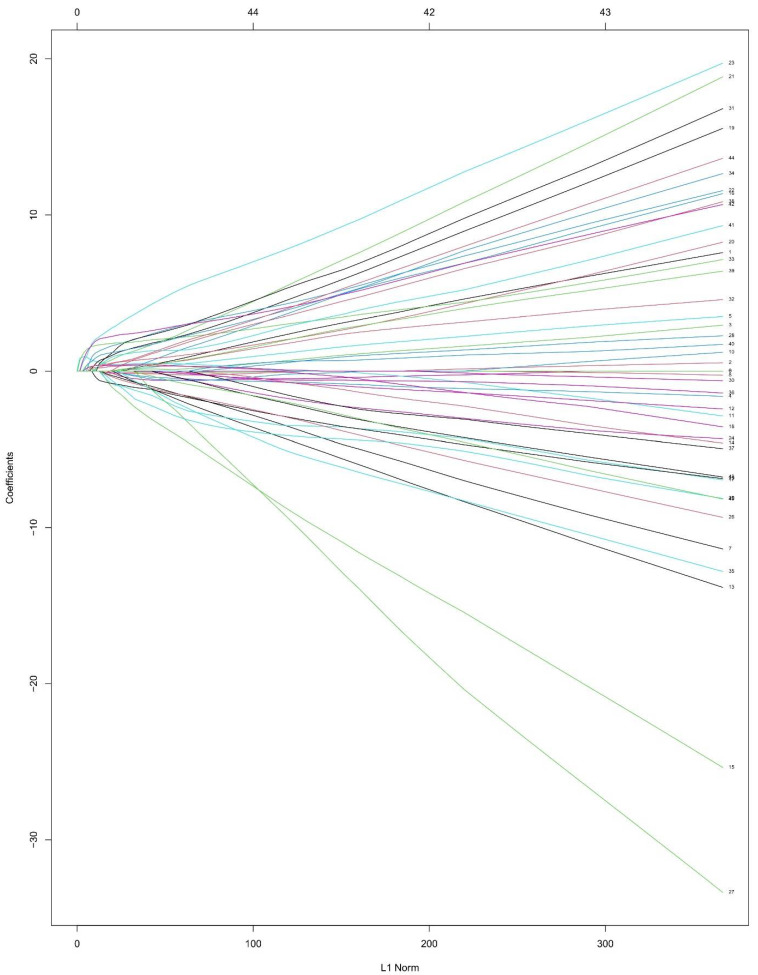
Plot for LASSO regression. The coefficients are measured on the y−axis. The penalty term is measured on the x−axis. Every colorful line represents the change in a variable in the regression. As the penalty term increased, the coefficients of most variables were shrunk to zero.

**Figure 3:**
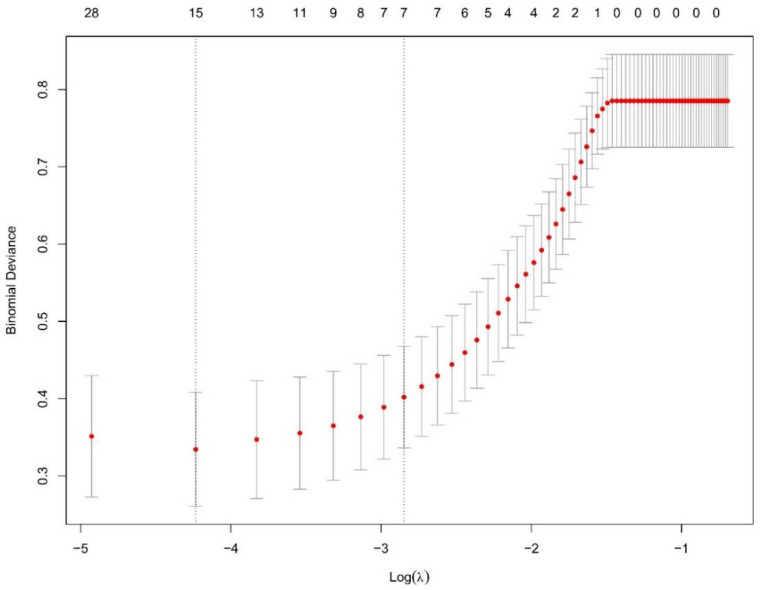
Plot for cross-validation of the penalty term. The *y*-axis measured the binomial deviance. The *x*-axis measured the log(λ). Two λ were selected after threefold cross-validation (corresponding to the two dotted lines on the graph). The former selected 15 variables and the latter selected 7 variables.

After converting multiple categorical variables to dummy variables, 45 variables were included in the LASSO regression analysis (Figure 2). The λ was selected by using a threefold cross-validation (Figure 3). There were two λ outputted, with one (former line, fifteen variables) representing the minimum binomial deviance and the other (latter line, seven variables) representing the largest λ that was still within a standard error (SE) of the minimum binomial deviance. The latter λ was selected since it resulted in a stricter limitation to decrease the number of variables than the former λ. Seven variables, including ARL, ascites level III, A/G, CREA, HDL, CA-125, and PIIIPN-P, were selected in the end according to the regression analysis (Table 2).

### 3.3. Fitted Model and Constructed Nomogram

**Figure 4:**
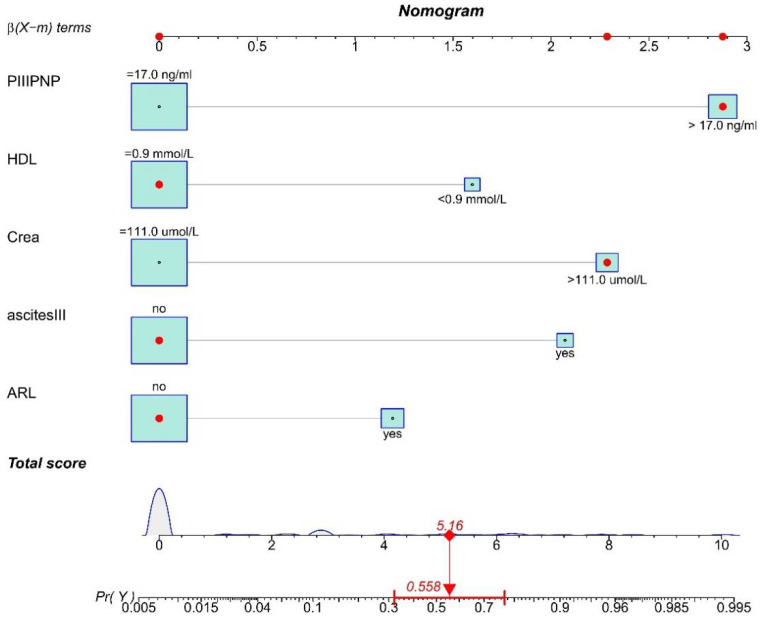
Nomogram for predicting the death probability of patients. The value of the top scale line is found corresponding to each independent variable, and then they are summed up. The value is projected onto the total score scale to present the corresponding death probability. PIIIPN-P, procollagen III N-terminal peptide; HDL, high-density lipoprotein; CREA, creatinine; ascites III, ascites level III; ARL, atrophy of the right liver.

We used the seven independent variables to construct a logistic model. According to the results shown in Table 3, two independent variables (X30, X39) were excluded for further analysis due to having *p* values greater than 0.05. Then, we fitted the model using the five independent variables (Table 4), including ARL(X11), ascites level III (X23), CREA (X33), HDL (X34), and PIIIPNP (X42). The nomogram for prognosis of advanced schistosomiasis was constructed according to the five predictors screened. Figure 4 showed an example of using the nomogram to predict the death probability of a given patient. The total score was determined based on summing up the individual scores calculated using the nomogram.

### 3.4. Assessment of Nomogram

**Figure 5:**
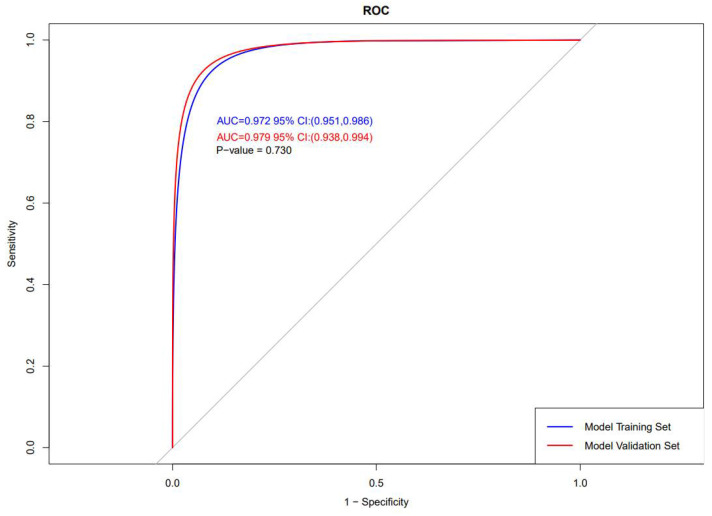
Plot for the ROC curves.

**Figure 6:**
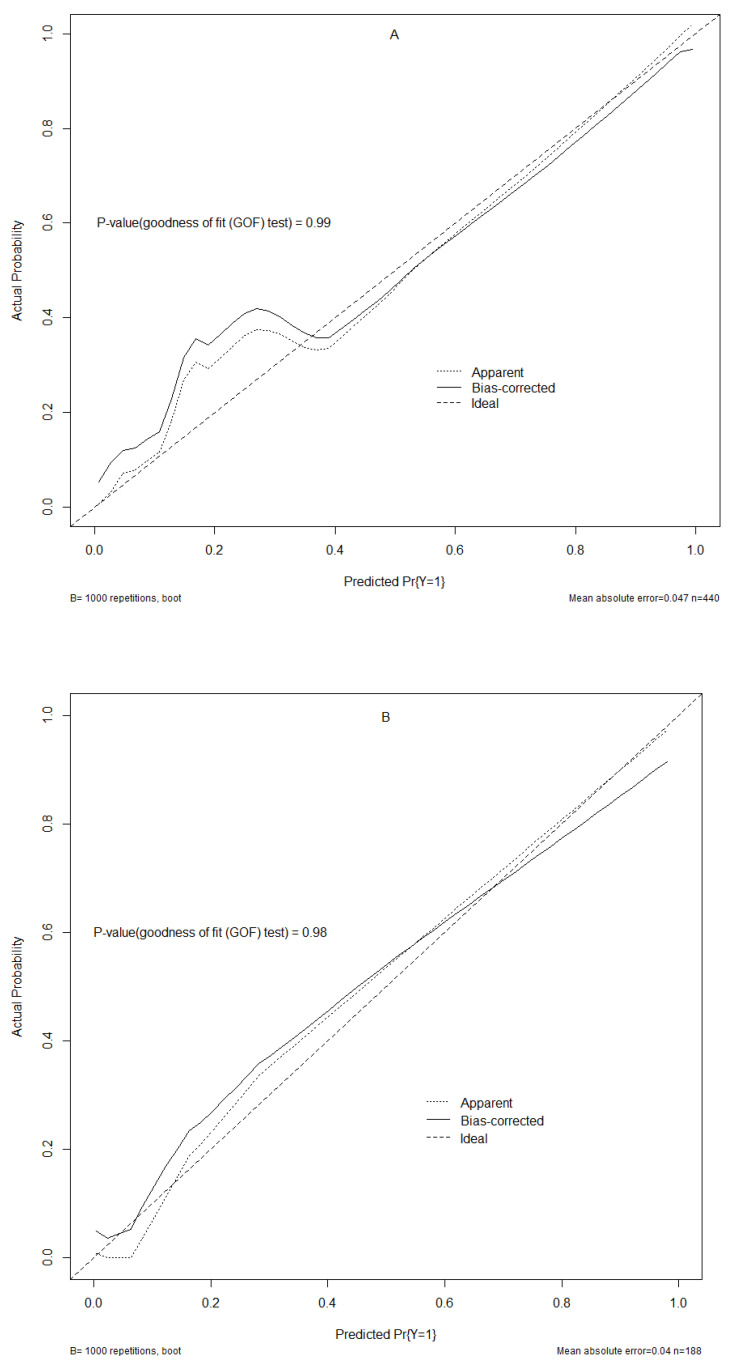
Plot for the calibration curves. (**A**): training set; (**B**): validation set. The ideal (fully fitted), bias-corrected (adjusted), and apparent (actual) curves were calculated by 1000 repetitions of bootstrapping samples. The *p* values for the training and validation sets’ GOF test were 0.99 and 0.98, respectively.

The nomogram’s performance is shown in Figure 5 and Figure 6. The result of the confusion matrix is shown in Supplementary Table S1. The C-index, sensitivity, specificity, PPV, and NPV of the nomogram were 0.97 (95% [CI]: [0.95–0.99]), 0.78 (95% [CI]: [0.64–0.87]), 0.97 (95% [CI]: [0.94–0.98]), 0.78 (95% [CI]: [0.64–0.87]), 0.97 (95% [CI]: [0.94–0.98]), respectively, in the training set; and 0.98 (95% [CI]: [0.94–0.99]), 0.86 (95% [CI]: [0.64–0.96]), 0.97 (95% [CI]: [0.93–0.99]), 0.79 (95% [CI]: [0.57–0.92]), 0.98 (95% [CI]: [0.94–0.99]), respectively, in the validation set, which showed an excellent ability to identify high-death-probability cases of this model. Meanwhile, after the bootstrap test for two ROC curves (*p* = 0.730), the model performance was not significantly different between the training and validation sets. The calibration curves of internal validation approached the ideal line, and the *p* values for the goodness of fit (GOF) test of training and validation sets were both greater than 0.95, which showed good consistency between the actual observations and predictive values calculated by the nomogram.

### 3.5. Clinical Use

**Figure 7:**
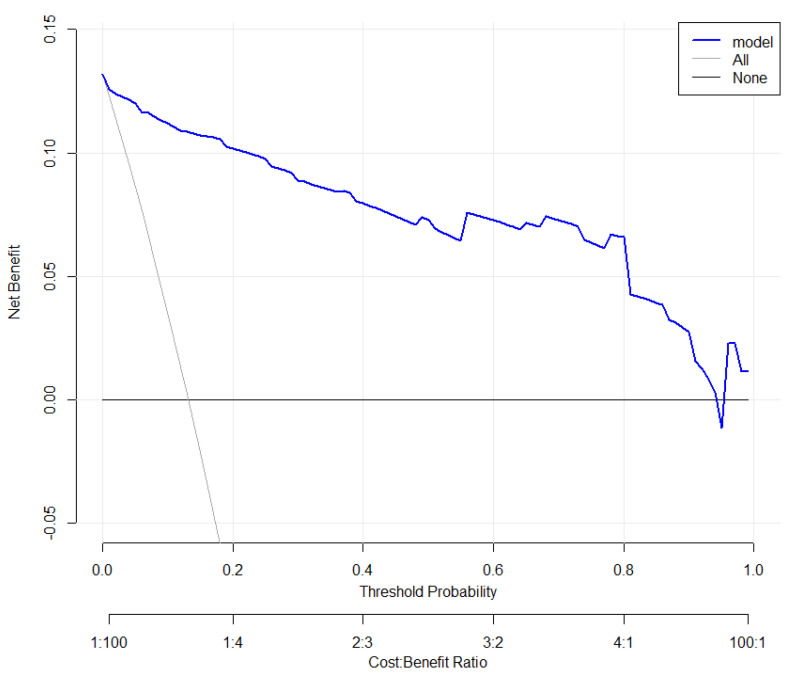
Plot for the clinical decision curve. The *y*-axis measures the net benefit. The blue line indicates the nomogram’s prediction. The thin gray line represents all patients for whom death would occur. The thick gray line represents no patients for whom death would occur. The net benefit was calculated by subtracting the proportion of all patients who were false positives from the proportion who were true positives, weighted by the relative harm of forgoing treatment compared to the negative consequences of unnecessary treatment.

**Figure 8:**
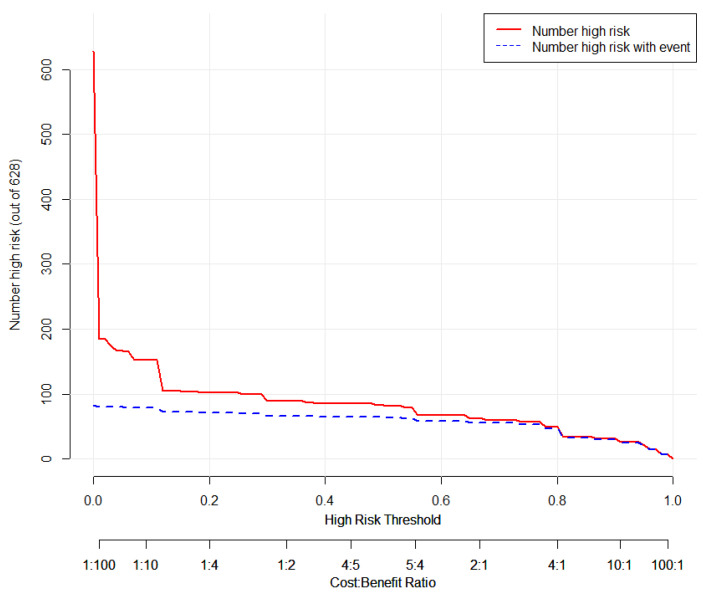
Plot for the clinical impact curve. The *y*-axis measures the number of patients with a high death probability. The red line represents the number of patients with high death probability predicted by the nomogram. The blue line represents the actual number of patients with high death probability.

The decision curve (Figure 7) showed that predicting death probability by the nomogram was more beneficial than the treat-none scheme or the treat-all-patients scheme. For example, if the patient chose treatment if their probability of death was 20% (the personal threshold probability of a patient is 20%), then the net benefit was 0.1. Physicians make decisions by the nomogram of whether implementing treatment has more benefit than the treat-none scheme or the treat-all-patients scheme. Furthermore, the clinical impact curve (Figure 8) shows the number of patients at death predicted by the nomogram and the actual number of patients at death under different threshold probabilities.

## 4. Discussion

The Chinese government gives high priority to advanced schistosomiasis due to its health and economic impact. Since 2005, the national schistosomiasis control program has assisted advanced schistosomiasis patients by providing subsidies to advanced cases for medical treatment [37]. According to Yang’s report, ascites and megalosplenia are the major subtypes of advanced schistosomiasis in China [38]. In our study, all patients with advanced schistosomiasis were diagnosed as a subtype of ascites and received medical assistance for advanced schistosomiasis treatment before our study. However, about 35 advanced cases die annually. Exploring the prognostic factors and predicting their prognosis could help clinicians identify individuals with a high risk of unfavorable prognoses requiring specific attention and interventions. 

We use retrospective cross-sectional research and LASSO logistic regression to explore the relationship between the prognostic outcomes of advanced schistosomiasis with population-based demographic, clinical, laboratory, and ultrasonic data. Prognostic factors were selected and used to construct a nomogram to predict death probabilities, including atrophy of the right liver, ascites level III, CREA, HDL, and PIIIPN-P. This model provides a plausible tool for clinical staff to screen advanced schistosomiasis patients with a high death probability, as well as a theoretical reference to plan treatment and decrease the disease burden of schistosomiasis [37]. 

In our study, 15.13% of patients (95/628) presented atrophy of the right liver, with 63 patients in the training set and 32 in the validation set. Previous studies have shown that advanced schistosomiasis cases with atrophy of the right liver typically featured a thickened wall of the portal vein branch of the right hepatic lobe in varying degrees, narrow blood lumen, slowed blood flow velocity, and a blocked right portal vein and its branches, without blood flow passing through [39,40]. Patients with right liver resection undergo persistent thrombocytopenia [41,42] and protein synthesis disorders [43], which may result in other complications, such as gastrointestinal bleeding and hypoalbuminemia (which is similar to this study). We found that patients with atrophy of the right liver had higher risks of coagulation disorders and hypoalbuminemia than the usual (*p* < 0.01). Liver disease, especially cirrhosis, is characterized by reduced synthesis of procoagulant proteins [44], which may lead to spontaneous bleeding [45] and varicose vein rupture (the most severe forms of bleeding in liver cirrhosis [46]). Some patients with cirrhosis might occur coagulation imbalance due to relevant anticoagulant protein deficiency and coagulation factor excess. The coagulation imbalance in some patients with cirrhosis is due to relevant anticoagulant protein deficiency and coagulation factor excess. Some patients are prone to hypercoagulation [45], which may result in deep vein thrombosis (DVT) and even disseminated intravascular coagulation (DIC). If the thrombosis falls off, pulmonary embolism may occur, which is fatal to patients. Furthermore, hypoalbuminemia is associated with a hypercatabolic state [47] and low synthetic ability, leading to excessive protein loss. Moreover, it also reflects that these patients are malnutritional, and there are not sufficient nutrients to be used for protein synthesis [48]. That is a vicious circle, and long-term hypoalbuminemia may aggravate the degree of liver damage [49], increase the risk of acute infection, and thus decrease the lifespan of patients. Therefore, we suppose that the atrophy of the right liver results from severe liver disease, as the liver has lost most of its functions at this time. However, in previous studies of advanced schistosomiasis, atrophy of the right liver did not receive adequate attention. We need to conduct additional studies to explore the prognosis of advanced schistosomiasis with atrophy of the right liver.

Ascites is the excessive fluid accumulation in the peritoneal cavity, which is also the most common symptom of advanced hepatic disease. As the dominant complication of liver-specific damage, the severity of ascites directly affects the overall prognosis. The survival rate of cirrhosis with ascites reaches only 60% within one year, while in refractory ascites cases, the six-monthly survival rate does not exceed 50% [50]. The presence of severe ascites is one of the strongest predictors of an elevated disability level in advanced schistosomiasis patients [14]. Consistent with these previous studies [51] of forecasting or predicting the prognosis of advanced schistosomiasis, severe ascites was also included in the nomogram model as a prognostic factor in our study. Mechanisms involved in ascites formation are portal hypertension, hypoalbuminemia, overproduction of fluid, or lymphatic obstruction [2]. The most common causes are liver cirrhosis, cancers, or heart failure [52]. It is a chronic wasting disease that will decrease the patients’ overall quality of life [53]. As the disease deteriorates, it is easy to have bacterial peritonitis (which will result in long-term fever and even severe infection) or acute heart failure. Furthermore, massive ascites can produce abdominal discomfort, such as abdominal swelling, pain, anorexia, and fatigue [54,55]. Moreover, massive ascites also can hinder mobility [56] and damage the personal appearance of the patients, which will increase the patient’s boredom and decrease their life quality [57]. Unfortunately, not all patients with advanced schistosomiasis are diagnosed in time to receive effective treatment.

In addition, this study selected CREA, HDL, and PIIIPN-P as independent predictors of mortality risk among various biochemical variables. An increasing serum creatinine concentration indicated decreased glomerular filtration, which reflects that the kidney may have been damaged [58]. It was easy for patients with long-term impaired renal function to suffer water-sodium retention (induced acute heart failure), hypoalbuminemia (led to refractory ascites), and hyperazotemia (led to hepatic encephalopathy) [59,60]. PIIIPN-P increased at the early and later stages of liver fibrosis, which is an indicator of active liver fibrosis [46,61], and indicated the degree of liver fibrosis deterioration. HDL was the smallest and densest of all lipoprotein classes [62], affecting cholesterol export from macrophages. Furthermore, it plays a vital anti-inflammatory, antioxidant, and antithrombotic role [63,64], enhancing endothelial repair, improving endothelial function, and suppressing leukocyte production in bone marrow [65,66]. Patients with advanced schistosomiasis with long-term, low-level HDL indicated that their metabolism disorder was severe and the liver or kidney might have been damaged. Differing from previous reports [67,68], hyaluronic acid (HA), an indicator reflecting the degree of liver fibrosis, was not included in our model based on LASSO regression, which the difference in the subtype of advanced schistosomiasis and cohort population might explain.

The prognosis of patients with advanced schistosomiasis is influenced by many factors. Traditional prediction models, such as the COX proportional hazards model or simple logistic model, usually present bad performance because these methods cannot cope well with linear, nonlinear, and multicollinear relationships. In addition, overfitting should be avoided to increase discriminative ability. In this study, we first introduce the LASSO logistic model to predict the death probability of advanced schistosomiasis patients. The advantages of this model are that it can process hundreds of factors for predicting patients’ prognosis to minimize multicollinearity and avoid overfitting among variables. From the results, the nomogram developed in our study performed excellent discriminative ability with a C-index higher than 0.97 both in the training set and validation set. The sensitivity (0.78 and 0.86 in the training and validation sets) was lower than the specificity (0.97 in the training and validation sets) and C-index. That could be because the ratio of positive outcomes in the overall sample was low (0.13). It could result in the model not being further trained and a limited ability to identify patients with a probability of death between 0.5 and 0.6. DCA proved that our nomogram predicted death probability with good clinical benefit and utility. The nomogram developed in our study provides a plausible tool for clinics to screen advanced schistosomiasis patients at high risk of death. It also provides a theoretical reference for improving China’s treatment and assistance programs for patients with advanced schistosomiasis japonica.

There are several limitations of this research. One limitation is that ascites is the only subtype of advanced schistosomiasis in Dongzhi County, and whether the model could be used for other subtypes is unknown. Another limitation is that the performance of the developed nomogram was only assessed by internal validation. Further prospective studies expanded to other subtypes of advanced schistosomiasis, other regions, and a larger sample should be conducted further to validate and optimize the nomogram that we developed.

## Figures and Tables

**Table 1:** Characteristics of patients with advanced schistosomiasis in this study.

Variables	Assigned Variable	Categories	Training Set	Validation Set	*p*-Value
outcome ^#^	Y	0 = live	382 (87%)	164 (87%)	0.99
		1 = death	58 (13%)	24 (13%)	
age ^#^	X1	0 = younger than or equal to 65	196 (45%)	86 (46%)	0.85
		1 = older than 65	244 (55%)	102 (54%)	
gender ^#^	X2	0 = male	185 (42%)	94 (50%)	0.08
		1 = female	255 (58%)	94 (50%)	
cholecystectomy ^#^	X3	0 = no	374 (85%)	155 (82%)	0.49
		1 = yes	66 (15%)	33 (18%)	
splenectomy ^#^	X4	0 = no splenectomy	271 (62%)	101 (54%)	0.08
		1 = splenectomy	169 (38%)	87 (46%)	
hypertension ^#^	X5	0 = no	302 (69%)	136 (73%)	0.35
		1 = yes	138 (31%)	51 (27%)	
hypoalbuminemia ^#^	X6	0 = no	335 (76%)	135 (72%)	0.30
		1 = yes	105 (24%)	53 (28%)	
hypokalemia ^#^	X7	0 = no	380 (86%)	165 (88%)	0.73
		1 = yes	60 (14%)	23 (12%)	
gastrointestinal bleeding ^#^	X8	0 = no	417 (95%)	176 (94%)	0.70
		1 = yes	23 (5%)	12 (6%)	
coagulopathy ^#^	X9	0 = no	321 (73%)	143 (76%)	0.48
		1 = yes	119 (27%)	45 (24%)	
liver fibrosis level ^#^	X10	0 = II level	383 (87%)	161 (86%)	0.73
		1 = III level	57 (13%)	27 (14%)	
ARL ^#^	X11	0 = no	377 (86%)	156 (83%)	0.46
		1 = yes	63 (14%)	32 (17%)	
gallbladder disease ^#^	X12	0 = no	300 (68%)	127 (68%)	0.95
		1 = yes	140 (32%)	61 (32%)	
diabetes ^#^	X13	0 = no	411 (93%)	173 (92%)	0.65
		1 = yes	29 (7%)	15 (8%)	
HBV infection ^#^	X14	0 = no	428 (97%)	183 (97%)	1 ^&^
		1 = yes	12 (3%)	5 (3%)	
hepatic encephalopathy ^#^	X15	0 = no	436 (99%)	186 (99%)	1 ^&^
		1 = yes	4 (1%)	2 (1%)	
other cancer ^#^	X16	0 = no	432 (98%)	184 (98%)	0.76
		1 = yes	8 (2%)	4 (2%)	
occupation ^#^	ref	0 = farmer	409 (93%)	176 (93%)	0.94
	X17	1 = fisher	2 (1%)	1 (1%)	
	X18	1 = other	29 (6%)	11 (6%)	
anemia level ^#^	ref	0 = normal	326 (74%)	145 (77%)	0.81
	X19	1 = I level	71 (16%)	29 (15%)	
	X20	1 = II level	34 (8%)	12 (6%)	
	X21	1 = III level	9 (2%)	2 (1%)	
ascites level ^#^	ref	0 = I level	362 (82%)	151 (80%)	0.65
	X22	1 = II level	43 (10%)	23 (12%)	
	X23	1 = III level	35 (8%)	14 (8%)	
AST/ALT ^#^	ref	0 = 1.0 to 1.2	63 (14%)	24 (13%)	0.21
	X24	1 = less than 1.0	66 (15%)	39 (21%)	
	X25	2 = greater than or equal to 1.2	311 (71%)	125 (66%)	
ALB ^#^	ref	0 = 36.0 to 55.0 g/L	246 (56%)	103 (55%)	0.97
	X26	1 = less than 36.0 g/L	183 (42%)	80 (42%)	
	X27	2 = greater than or equal to 55.0 g/L	11 (2%)	5 (3%)	
TP ^#^	ref	0 = 65.0 to 85.0 g/L	219 (50%)	79 (42%)	0.17
	X28	1 = less than 65.0 g/L	190 (43%)	91 (48%)	
	X29	2 = greater than or equal to 85.0 g/L	31 (7%)	18 (10%)	
A/G ^#^	ref	0 = 1.0 to 2.5	399 (91%)	175 (93%)	0.66
	X30	1 = less than 1.0	36 (8%)	12 (6%)	
	X31	2 = greater than or equal to 2.5	5 (1%)	1 (1%)	
CREA ^#^	ref	0 = 57.0 to 111.0 umol/L	346 (79%)	144 (77%)	0.70
	X32	1 = less than 57.0 umol/L	35 (8%)	14 (7%)	
	X33	2 = greater than or equal to 111.0 umol/L	59 (13%)	30 (16%)	
HDL ^#^	ref	0 = 0.9 to 2.0 mmol/L	400 (91%)	177 (94%)	0.39
	X34	1 = less than 0.9 mmol/L	32 (7%)	8 (4%)	
	X35	2 = greater than or equal to 2.0 mmol/L	8 (2%)	3 (2%)	
BMI ^#^	ref	0 = 18.5 to 23.9	263 (60%)	110 (58%)	0.75
	X36	1 = less than 18.5	71 (16%)	26 (14%)	
	X37	2 = 23.9 to 27.9	86 (20%)	43 (23%)	
	X38	3 = greater than or equal to 27.9	20 (4%)	9 (5%)	
CA125 ^#^	X39	0 = less than or equal to 35.0 KU/L	315 (72%)	130 (69%)	0.60
		1 = greater than 35.0 KU/L	125 (28%)	58 (31%)	
HA ^#^	X40	0 = less than or equal to 106.0 ng/mL	209 (48%)	80 (43%)	0.29
		1 = greater than 106.0 ng/mL	231 (52%)	108 (57%)	
LN ^#^	X41	0 = less than or equal to 133.0 ng/mL	429 (97%)	185 (98%)	0.57
		1 = greater than 133.0 ng/mL	11 (3%)	3 (2%)	
PIIIPN-P ^#^	X42	0 = less than or equal to 17.0 ng/mL	350 (80%)	148 (79%)	0.90
		1 = greater than 17.0 ng/mL	90 (20%)	40 (21%)	
CIV ^#^	X43	0 = less than or equal to 98.0 ng/mL	306 (70%)	120 (64%)	0.19
		1 = greater than 98.0 ng/mL	134 (30%)	68 (36%)	
TBIL ^#^	X44	0 = less than or equal to 19 umol/L	310 (70%)	136 (72%)	0.70
		1 = greater than 19.0 umol/L	130 (30%)	52 (28%)	
DBIL ^#^	X45	0 = less than or equal to 6.8 umol/L	289 (66%)	127 (68%)	0.72
		1 = greater than 6.8 umol/L	151 (34%)	61 (32%)	

ARL, atrophy of the right liver; AST/ALT, the ratio of aspartate aminotransferase and alanine aminotransferase; ALB, albumin; TP, total protein; A/G, the ratio of albumin and globulin; CREA, creatinine; HDL, high-density lipoprotein; BMI, body mass index; CA125, CA-125 antigen; HA, hyaluronic acid; LN, laminin; PIIIPN-P, procollagen III N-terminal peptide; CIV, IV collagen; TBIL, total bilirubin; DBIL, direct bilirubin. ^#^ Frequency and proportion; chi-square test was used to compare differences between groups. ^&^ the value was close to 1.

**Table 2:** The coefficients screened by the LASSO regression.

Variables	Coefficient	Variables	Coefficient	Variables	Coefficient
(Intercept)	−3.029813	X16	.	X32	.
X1	.	X17	.	X33	1.2633524
X2	.	X18	.	X34	0.1707879
X3	.	X19	.	X35	.
X4	.	X20	.	X36	.
X5	.	X21	.	X37	.
X6	.	X22	.	X38	.
X7	.	X23	0.778357	X39	0.2489765
X8	.	X24	.	X40	.
X9	.	X25	.	X41	.
X10	.	X26	.	X42	0.9806545
X11	0.913022	X27	.	X43	.
X12	.	X28	.	X44	.
X13	.	X29	.	X45	.
X14	.	X30	0.1122175		
X15	.	X31	.		

“.”: coefficients were shrunk to zero by regularization. X11, ARL; X23, ascites level III; X30, A/G; X33, CREA; X34, HDL; X39, CA-125; X42, PIIIPN-P.

**Table 3:** Model of all independent variables.

	Estimate	Std. Error	Z Value	*p* Value	Exp (Estimate)	Exp (Estimate) 95% CI
(Intercept)	−5.119	0.580	−8.834	<0.001 ***	0.006	(0.002–0.016)
X11	1.063	0.538	1.976	0.048 *	2.894	(0.991–8.273)
X23	1.723	0.661	2.606	0.009 **	5.599	(1.579–21.618)
X30	0.356	0.675	0.527	0.598	1.428	(0.382–5.509)
X33	2.057	0.516	3.986	<0.001 ***	7.825	(2.883–22.133)
X34	1.446	0.724	1.997	0.046 *	4.244	(1.048–18.350)
X39	0.922	0.654	1.409	0.159	2.514	(0.709–9.596)
X42	2.445	0.594	4.116	<0.001 ***	11.532	(3.761–39.682)

Significance codes: *** 0.001; ** 0.01; * 0.05. X11, ARL; X23, ascites level III; X30, A/G; X33, CREA; X34, HDL; X39, CA-125; X42, PIIIPN-P.

**Table 4:** Model of five independent variables.

	Estimate	Std. Error	Z Value	*p* Value	Exp (Estimate)	Exp (Estimate) 95% CI
(Intercept)	−4.930	0.534	−9.234	<0.001 ***	0.007	(0.002–0.018)
X11	1.191	0.530	2.248	0.025 *	3.290	(1.146–9.276)
X23	2.071	0.632	3.278	0.001 **	7.936	(2.369–28.975)
X33	2.286	0.492	4.645	<0.001 ***	9.838	(3.802–26.572)
X34	1.598	0.666	2.401	0.016 *	4.942	(1.377–19.168)
X42	2.876	0.537	5.353	<0.001 ***	17.749	(6.527–55.271)

Significance codes: *** 0.001; ** 0.01; * 0.05. X11, ARL; X23, ascites level III; X33, CREA; X34, HDL; X42, PIIIPN-P.

## Data Availability

Data are not available due to ethical restrictions.

## Supplementary Materials

The following supporting information can be downloaded at: https://www.mdpi.com/article/10.3390/tropicalmed8010033/s1, Figure S1: Dongzhi County; Table S1: Results of the confusion matrix.
