# Supplementary material for: A Nomogram for Predicting Prognosis of Advanced Schistosomiasis japonica in Dongzhi County—A Case Study
Source: Trop Med Infect Dis. 2023 Jan 3;8(1):33. doi: 10.3390/tropicalmed8010033 (PMC9866143; doi:10.3390/tropicalmed8010033)
Supplement: Supplementary file 1 [file tropicalmed-08-00033-s001.zip › tropicalmed-2015742-supplementary.pdf]

Tropical Medicine and Infectious Disease

Supplementary Materials for A nomogram for predicting prognosis of advanced schistosomiasis japonica in Dongzhi County-a case study

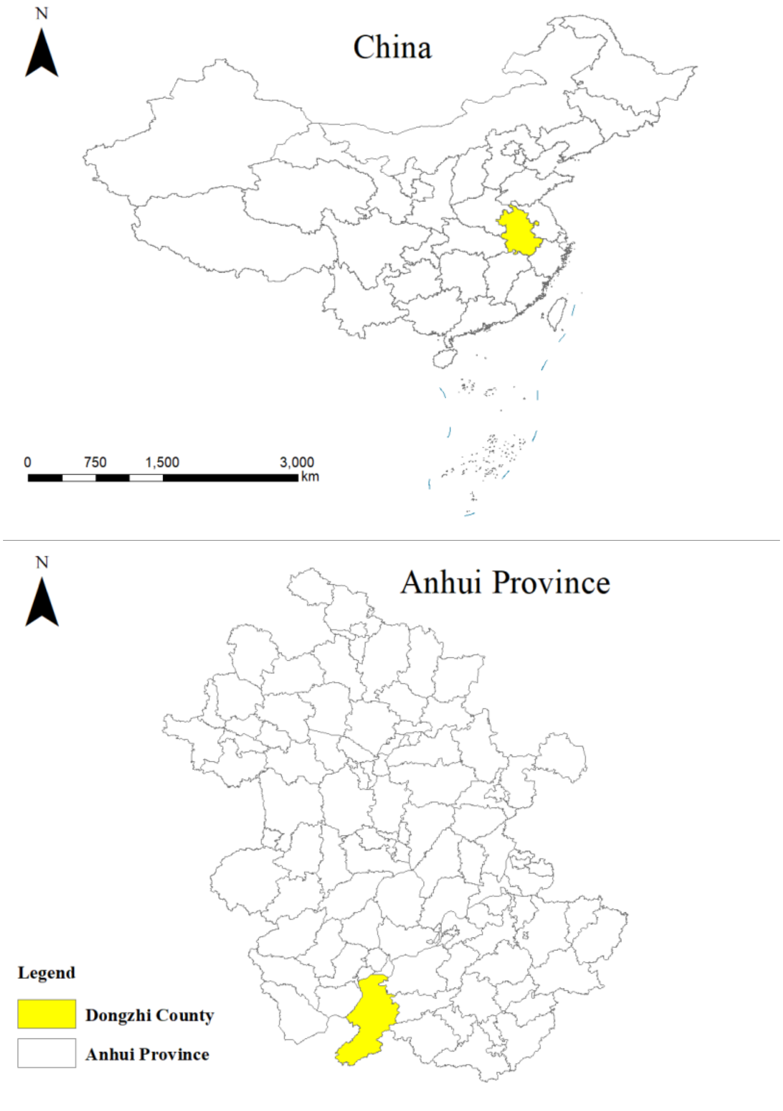

Figure S1. Dongzhi County.

Table S1. Results of the confusion matrix.

		Training set		Validation set	
		Actual		Actual	
		Positive	Negative	Positive	Negative
Predictive	Positive	45	13	19	5
	Negative	13	369	3	161
